# Specificity of the AMP-6000 Method for Enumerating *Clostridium* Endospores in Milk

**DOI:** 10.3390/foods13081192

**Published:** 2024-04-13

**Authors:** Johanna Burtscher, Tamara Rudavsky, Ulrike Zitz, Konrad J. Domig

**Affiliations:** 1 Institute of Food Science, Department of Food Science and Technology, BOKU University, Muthgasse 18, 1190 Vienna, Austriakonrad.domig@boku.ac.at (K.J.D.); 2 Austrian Competence Centre for Feed and Food Quality, Safety and Innovation (FFoQSI), Technopark 1D, 3430 Tulln, Austria

**Keywords:** *Clostridium tyrobutyricum*, milk quality, endospores, most probable number, *Paenibacillus*, *Bacillus*, cheese spoilage

## Abstract

Enumeration of endospores of butyric acid-forming clostridia in cheese milk is an essential part of milk quality monitoring for cheese producers to avoid late blowing, severe spoilage caused by clostridia during ripening. However, due to the lack of an internationally standardized method, different methods are used and it is important to consider how the choice of method affects the results. This is particularly relevant when clostridial spore counts in milk are considered for quality payments. The aim of this study was to evaluate the specificity of the AMP-6000 method for the enumeration of endospores of cheese spoiling clostridia in milk. First, to assess the prevalence of *Clostridium* diversity and to determine potential non-target species, we identified isolates from positive reactions of the AMP-6000 method used to quantify clostridial endospores in raw milk and teat skin samples by MALDI-TOF MS. Based on these results, a strain library was designed to evaluate method inclusivity and exclusivity using pure cultures of target and non-target strains according to ISO 16140-2:2016. Most target *Clostridium tyrobutyricum* strains, as well as all tested *C. butyricum* and *C. sporogenes* strains were inclusive. However, *C. beijerinckii* may be underestimated as only some strains gave positive results. All non-target strains of bacilli and lysinibacilli, but not all paenibacilli, were confirmed to be exclusive. This study provides performance data to better understand the results of microbiological enumeration of butyric acid-forming clostridia in milk and serves as a basis for future methodological considerations and improvements.

## 1. Introduction

Enumerating endospores of butyric acid-forming clostridia in cheese milk is an essential part of raw milk quality monitoring for hard and semi-hard cheese producers to avoid late blowing, severe spoilage caused by clostridia during ripening. Clostridia are able to metabolize lactate, thereby producing excessive amounts of gas and butyric acid, which cause undesired openings and cracks as well as an unpleasant aroma and off-flavors in cheese [1]. The four main clostridial species associated with late blowing in cheese are the saccharolytic *Clostridium tyrobutyricum*, *C. butyricum* and *C. beijerinckii*, as well as the proteolytic *C. sporogenes* [2]. Careful monitoring of raw milk quality and prevention of cheese milk contamination with clostridial endospores can reduce significant financial losses and food waste due to product spoilage. Therefore, some countries have integrated the detection and enumeration of clostridial endospores in milk into the routine quality assessment of raw milk [3]. However, the enumeration of butyric acid-producing clostridia in raw milk remains a challenge. It is known that extremely low numbers of clostridial spores can cause severe spoilage in hard cheeses [1,4]. Therefore, methods with low detection limits of a few spores per liter of raw milk are required. For this reason, most probable number (MPN) methods, although labor-intensive, are widely used to quantify clostridial endospores in raw milk. The conventional detection principle is usually based on the detection of gas as a consequence of outgrowth and germination of clostridial spores [1]. However, insufficient medium selectivity has been observed when conventional MPN procedures to enumerate clostridial endospores were evaluated [5].

Brändle et al. [6] optimized an adapted medium in combination with a semi-automated MPN application for the enumeration of clostridial endospores in milk, the now commercially available AMP-6000 method. The method consists of a miniaturized MPN procedure in which a mixture of pasteurized milk sample and ready-to-use medium (AMPMedia666) is distributed in 96-well plates, followed by detection of clostridial growth after 48 h of anaerobic incubation by detecting the change of a color indicator from red to yellow using a customized scanning device [3,6].

In a previous study, inclusivity tests were performed on the developed medium formulation. Inclusivity studies “involve pure target strains to be detected or enumerated by the alternative method” [7]. Three replicates of miniaturized mixtures of spiked milk-media samples were prepared with a maximum vessel volume of 350 µL per replicate [6]. Growth of a wide range of pure cultures of *Clostridium* spp. in AMPMedia666 and a high selectivity of the AMP-6000 method for clostridia were reported [6]. However, growth in the adapted medium was tested without considering the entire AMP-6000 analytical procedure, including larger sample volumes, pasteurization and standardized incubation. In addition, the inoculum concentration was not defined and pure cultures were not assessed simultaneously with a reference method [6]. Finally, to the best of our knowledge, data from exclusivity studies on the growth and enumeration of non-target strains, which may potentially cross-react in the AMP-6000 method, are not yet available [7].

The first aim of this study was to further evaluate the specificity of the AMP-6000 method for the enumeration of endospores of butyric acid-producing clostridia under practical conditions. For this purpose, isolates obtained from a large number of raw milk and teat skin samples were identified by matrix-assisted laser desorption/ionization time-of-flight mass spectrometry (MALDI-TOF MS) [8]. The milk samples and teat swab samples were collected and analyzed using the AMP-6000 method during a two-year monitoring study in eight Austrian dairy farms [8].

The identification of isolates obtained from positive reactions of the AMP-6000 method should allow conclusions to be drawn about the prevalence of *Clostridium* diversity in raw milk and on teat skin and, indirectly, about the specificity of the method. In addition, information on isolate diversity in these field samples analyzed using the AMP-6000 method should serve as a basis for the strain selection for further method performance testing.

The second aim of this study was a thorough evaluation of the specificity of the AMP-6000 method using pure bacterial cultures, based on the recommendations provided in ISO 16140-2:2016 “Method validation Part 2: Protocol for the validation of alternative (proprietary) methods against a reference method” [9]. However, although not recommended in the standard, the sample matrix milk was included in the specificity testing because it is considered not only as a sample, but also as a substantial part of the growth medium, as milk and medium are mixed in the AMP-6000 method at a ratio 3:1. The second study design challenge was the lack of a suitable official reference method with comparable specificity. An overview and comparison of the most commonly used MPN methods used for clostridial enumeration in milk is provided in more detail elsewhere [3,5]. Since previous studies had selected the so-called “Bryant and Burkey (BB) method” or “CNERNA” method as the reference method for comparison with the AMP-6000 method [3,6,10], we also selected the BB method as the reference method in the present study and used reinforced clostridial agar (RCA) as the non-selective agar. Quantitative growth results of all strains were obtained to facilitate data interpretation based on previous findings that the AMP-6000 method yielded significantly lower spore counts than the BB method when analyzing identical raw milk samples [6,10].

In this way, this study should provide important performance data for a better understanding of the results of microbiological enumeration of endospores of butyric acid-producing clostridia in raw milk. It should also serve as a basis for future methodological considerations and improvements.

## 2. Materials and Methods

### 2.1. Identification of Isolates from Raw Milk and Teat Skin Samples

The AMP-6000 method was used in a study to quantify clostridial endospores from a large number of milk samples. In addition, we included isolates from the analysis of teat skin swabs to challenge the method with a matrix containing high levels of endospore-forming bacteria. Field samples were collected from eight Austrian dairy farms during five seasons in 2018 and 2019, as described by Burtscher et al. [8]. The samples included pooled quarter milk (PQM; *n* = 631) from individual cows, bulk tank milk (BTM; *n* = 40) from each farm and teat swab samples (TS; *n* = 382). All samples were analyzed using the AMP-6000 method as previously described [8]. Smears on reinforced clostridial agar (RCA; Merck, Darmstadt, Germany) were prepared from randomly selected positive wells of incubated microtiter plates of the AMP-6000 method and incubated anaerobically at 37 °C. Grown colonies were identified by MALDI-TOF MS after sample preparation according to the manufacturer’s extended direct transfer procedure (Bruker Daltonics, Bremen, Germany). Spectra were acquired using a Microflex LT mass spectrometer (Bruker Daltonics, Bremen, Germany) according to the standard settings recommended by the manufacturer (method “MBT_AutoX”) and identified using the Bruker MBT database, the BIOTECTON Diagnostics D-Mass-02 database and an in-house database containing some additional *C. tyrobutyricum* spectra. If identification was not possible, the analysis was repeated using the formic acid extraction procedure (Bruker Daltonics, Bremen, Germany) as described in [11]. Clostridial species names of isolates that yielded a score > 1.7 in MALDI-TOF MS identification were considered for the presentation of results in the present study, based on experience that identification scores of spore-forming clostridia are sometimes lower than 2.0.

### 2.2. Strain Selection for Inclusivity and Exclusivity Testing

Method validation according to ISO 16140-2:2016 requires at least 50 target microorganisms and 30 non-target microorganisms [9]. The AMP-6000 method targets endospore-forming members of the genus *Clostridium*, with *C. tyrobutyricum* as the main target organism. Therefore, the majority of target test strains in the present study consisted of *C. tyrobutyricum* (*n* = 45). *C. sporogenes*, *C. butyricum* and *C. beijerinckii* were isolated from various late blown cheeses and, therefore, we also focused on these strains [1,5,12,13,14,15]. In addition, we selected other *Clostridium* species, which had been isolated from the field samples analyzed in the course of this study or identified in milk or cheese in previous studies [5,6,12,15]. In addition, because they have often been isolated from milk and at least some strains of these species appear to be capable of gas production [16,17], we included some former clostridia, that have since been assigned to other genera: *Paraclostridium bifermentans*, *Paeniclostridium sordellii* and *Lacrimispora celerecrescens*. For the selection of non-target strains, we focused on aerobic or facultatively anaerobic spore-formers and species, which had been isolated from milk, cheese and teat skin, including the three main genera *Bacillus* (*n* = 22), *Paenibacillus* (*n* = 13) and *Lysinibacillus* (*n* = 2). For all endospore-forming bacteria, we included publicly available strains from culture collections and isolates from milk and cheese whenever possible, taking into account differences between strains from various sources. Finally, we also included lactic acid bacteria (LAB), which may be present in ripened cheese or during cheese processing, in our test strain set to evaluate whether high numbers of starter cultures or thermoduric bacteria of the accompanying microbiota might interfere with the enumeration of clostridia using the AMP-6000 method.

### 2.3. Inclusivity Testing with Pure Cultures of Spore-Forming Bacteria

**Figure 1:**
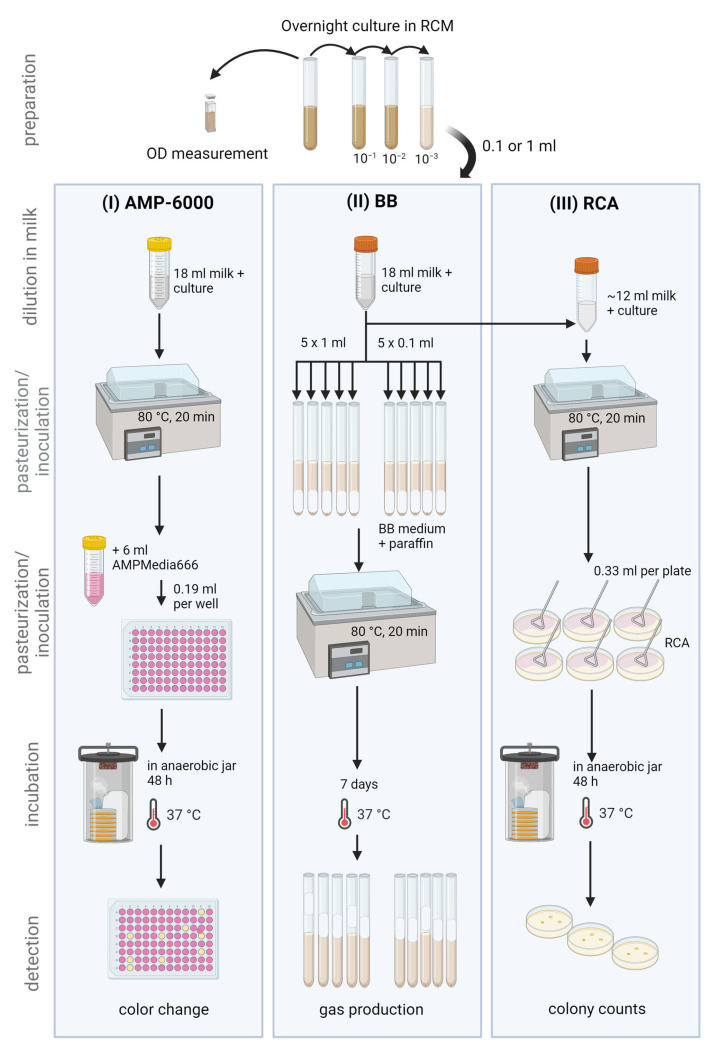
Strain activation in reinforced clostridial medium (RCM) and overview of the analytical procedure used to perform inclusivity testing of pure cultures of spore-forming bacteria using the (I) AMP-6000 method (alternative method) with AMPMedia666, (II) the BB method (reference method) using Bryant and Burkey medium and (III) reinforced clostridial agar (RCA) as a non-selective agar. Figure created with BioRender.com.

A total of 76 clostridial strains were used, including 45 *C. tyrobutyricum*, 24 other *Clostridium* species and 7 former *Clostridium* strains assigned to new genera (for detailed strain information see Table 1). Many of the strains analyzed derived from milk or cheese, and all strains tested were (re-)identified by MALDI-TOF MS and 16S rRNA gene sequencing prior to analysis. As milk is an essential part of the growth medium, extended shelf life (ESL) milk was spiked with the test strains to perform inclusivity tests. An overview of the analytical procedure is provided in Figure 1. The spiked milk samples were analyzed using the following three methods: (I) the AMP-6000 method (alternative method), (II) the BB method using Bryant and Burkey medium in glass tubes (reference method) and (III) the spread plate method on reinforced clostridial agar (RCA) (non-selective agar). All methods are described in more detail below. An inoculum concentration of 7300 spores/L of sample (determined by the AMP-6000 method) was aimed for, which is 100 times higher than the lowest limit of quantification of the AMP-6000 method (<73 spores/L of sample), according to the recommendations of ISO 16140-2:2016 [9]. All incubation steps were performed in anaerobic jars (atmosphere composition 80% N_2_, 10% CO_2_, 10% H_2_) using a jar gassing system (Don Whitley Scientific Limited, Bingley, UK). For the Bryant and Burkey method, the anaerobic atmosphere was created by sealing the tubes with a paraffin plug.

#### 2.3.1. Strain Activation for Inclusivity Testing

A loop of cell material was inoculated into RCM broth (Merck KGaA, Darmstadt, Germany) and incubated anaerobically at 37 °C until growth was visible (approximately 24–48 h). Cultures were then fractionated on RCA (Merck) and incubated at 37 °C for 48–72 h. Single colonies were inoculated into reinforced clostridial broth (RCM) and incubated for 24 h for inclusivity testing. For a few strains with limited sporulation, longer incubation times and a lower temperature of 30 °C were required to achieve sufficient growth. The optical density of the cultures (OD_600_) was measured to determine the need for decimal dilutions in buffered peptone water.

#### 2.3.2. Alternative MPN Method: AMP-6000

Based on the results of the OD measurement and preliminary experiments, the appropriate dilution (undiluted, 10^−1^, 10^−2^ or 10^−3^) and sample volume (1 mL or 0.1 mL) were selected for each test strain. Then, 17 mL or 17.9 mL of ESL milk (Milfina, 3.5% fat content, pasteurized and microfiltered, Hofer, Austria) was transferred to a sterile reaction tube (50 mL) with a custom-made filter element (SY-LAB Geräte GmbH, Neupurkersdorf, Austria) and either 1 mL or 0.1 mL of the undiluted or diluted overnight culture was added to reach a final volume of 18 mL. The mixture was then pasteurized in a water bath at 80 °C for 20 min. After cooling to approximately 50 °C, 6 mL of 4x-concentrated AmpMedia666 (SY-LAB) was added to the milk sample, and the milk-medium mixture was homogenized using a vortex mixer. Subsequently, 0.19 mL of the mixture was transferred into each well of a sterile 96-well plate using an AMP-6000-APS pipetting device (SY-LAB). The plates were capped and incubated in anaerobic jars for 48 h. Finally, the plates were evaluated using a scanning device and software that counts the number of positive reactions based on the change of the indicator dye from red to yellow and estimates the number of spores according to Hurley and Roscoe [18] (AMP-6000 Scan, SY-LAB). Clostridia were also quantified in each ESL milk package and a clostridial spore standard containing a known concentration of *C. tyrobutyricum* was periodically examined using the AMP-6000 method to evaluate and ensure correct method performance. The lower and upper detection limits of this AMP-6000 procedure were <73 and 32,000 spores/L of sample, respectively.

#### 2.3.3. Reference MPN Method: Bryant and Burkey

The same volumes of overnight culture and ESL milk as described above were prepared in a sterile reaction tube (50 mL) for each test strain. Approximately half the volume of the overnight culture in ESL milk was used as the inoculum for the BB method as previously described [6,19]. Briefly, 5 tubes containing 1 mL of sample and 5 tubes containing 0.1 mL of sample were prepared and sealed with a paraffin plug. The tubes were incubated at 37 °C for 7 days and positive reactions were indicated by a raised paraffin plug (no contact between medium and plug) due to gas production by clostridia. Based on the number of positive reactions per dilution step, the number of spores/L of sample was estimated using an MPN calculator [18]. The lower and upper detection limits of this MPN procedure were <180 and 16,000 spores/L of sample, respectively. The remaining overnight culture-milk mixture was used for non-selective plating.

#### 2.3.4. Non-Selective Method: Spread Plate on RCA

The milk-culture mixture in the reaction tube was pasteurized in a water bath at 80 °C for 20 min (same conditions as described above). After cooling, 1 mL of the mixture was divided and spread on three RCA plates in duplicate, resulting in a total of six inoculated RCA plates per test strain. After 48 h of incubation in an anaerobic jar at 37 °C, the grown colonies were counted. The lower and upper limits of this RCA procedure were <1000 and 900,000 spores/L sample, respectively.

### 2.4. Exclusivity Testing with Spore-Forming Bacteria and Non-Spore-Forming Bacteria

A total of 51 non-target strains of species that may be present in raw milk were selected for exclusivity testing. These strains included 37 spore-forming strains (22 bacilli, 2 lysinibacilli and 13 paenibacilli) and 14 non-spore-forming lactic acid bacteria (LAB) (detailed strain information available in Table 2 and Table 3). Non-spore-forming strains were also included in the exclusivity tests, mainly to evaluate whether the selected pasteurization conditions sufficiently inactivated potential accompanying microbiota even when present in high numbers, as is the case, for instance, when starter cultures have been added to the cheese milk or when cheese is analyzed.

#### 2.4.1. Strain Activation and Spore Preparation

Strains of the genera *Bacillus*, *Lysinibacillus* and *Paenibacillus* were streaked on nutrient agar (Merck) and incubated at 30 °C for 24 h. T3 agar plates [20] were inoculated with single colonies of each strain and incubated at 30 °C for 7 days. Sporulation was checked using phase contrast microscopy. Endospores were floated from T3 agar plates with 5 mL of physiological saline solution and transferred to a 50 mL reaction tube. Residual cells were harvested by rinsing the plates again with 2 mL saline solution and transferring them to the same 50 mL reaction tube. The reaction tubes were then centrifuged at 8000× *g* for 10 min at 4 °C. Bacterial cell pellets were stored at 4 °C until further analysis. Streaks of lactic acid bacteria were prepared on MRS agar (Merck) and incubated anaerobically for 4 days. An incubation temperature of 30 °C or 37 °C was chosen according to the recommendations of the culture collection for the respective strain. To obtain overnight cultures, single colonies were then transferred to tubes containing 4 mL of MRS broth and incubated for 24 h under the same conditions as before.

#### 2.4.2. Sample Preparation

Thawed spore pellets were centrifuged again at 8000× *g* for 10 min at 4 °C and the supernatant was discarded. The spores were then resuspended in 22 mL ESL milk (Milfina, longer fresh, 3.5% fat, pasteurized and microfiltered) and mixed thoroughly by inversion of the tube 25 times. Overnight cultures of lactic acid bacteria were centrifuged at 8000× *g* for 10 min at 4 °C. The supernatant was discarded and the cells were washed again with 4 mL of physiological saline. After another centrifugation step as described above, the supernatant was discarded and the cell pellet was resuspended in 1 mL ESL milk (as described above).

#### 2.4.3. Alternative Method: AMP-6000

A 20 mL sample of the spiked milk was transferred into a sterile 50 mL reaction tube with a custom filter element and pasteurized at 80 °C for 20 min. Next, 2 mL of the pasteurized sample was transferred to a sterile tube for the non-selective spread plate method and the remaining sample (18 mL) was processed as described in Section 2.3.2. 

#### 2.4.4. Reference Method: Bryant and Burkey

Three glass tubes were inoculated three times with 0.1 mL of sample (detection limit <4100 spores/L), sealed with paraffin, and pasteurized at 80 °C for 20 min. The analysis of two *Paenibacillus* strains (LMG 19079 and LMG 14832) was repeated because low numbers of cells and/or spores in phase contrast microscopy and weak growth in RCM indicated the possibility of low spore counts. For this purpose, another biological replicate consisting of fresh spore suspension was prepared, and higher inocula were used to achieve a lower detection limit (5 times 1 mL and 5 times 0.1 mL sample, resulting in a detection limit of <180 spores/L of sample). All tubes were incubated anaerobically at 37 °C for 7 days. Positive reactions were indicated by a raised paraffin plug (no contact between medium and plug) due to gas production and the MPN number was estimated using an MPN calculator [18].

#### 2.4.5. Non-Selective Spread Plate on RCA

Decimal dilutions of the (pasteurized or unpasteurized) samples were prepared with physiological saline solution and each dilution was spread on RCA in duplicates. One RCA plate was incubated anaerobically at 37 °C for 48 h (standard conditions to enumerate clostridia). Another plate was incubated at the optimal growth condition for bacilli, lysinibacilli and paenibacilli (30 °C, aerobic). Dilutions of the unpasteurized samples of LAB were spread on MRS instead of RCA and incubated at the suggested optimal condition for each strain suggested by the provider to determine initial inoculation volumes.

### 2.5. Statistical Analyses

When results were below or above the detection limits of the methods, the value of the lower or upper limit of each method was used for statistical analysis. Statgraphics Centurion V 19 was used for statistical analysis and results were considered significant at *p* < 0.05. Results were log_10_ transformed to obtain values that were as normally distributed as possible. The Shapiro–Wilk test showed a normal distribution only for the AMP-6000 results (0.95; *p* = 0.06), while the BB (0.95; *p* = 0.04) and RCA (0.87; *p* = 0.00) data were not normally distributed. Therefore, all data were analyzed using robust tests: Wilcoxon matched-pairs test to compare the alternative (AMP-6000) and the reference method (BB), and Friedman’s test to compare all three methods. Spearman’s correlations were estimated and the interpretation of effect size was based on Cohen [21], with *r* = 0.10, *r* = 0.30 and *r* = 0.50 considered small, medium and large, respectively.

## 3. Results and Discussion

### 3.1. Isolate Identification from Positive Reactions after the Analysis of Milk and Teat Skin Swab Samples Using the AMP-6000 Method

**Figure 2:**
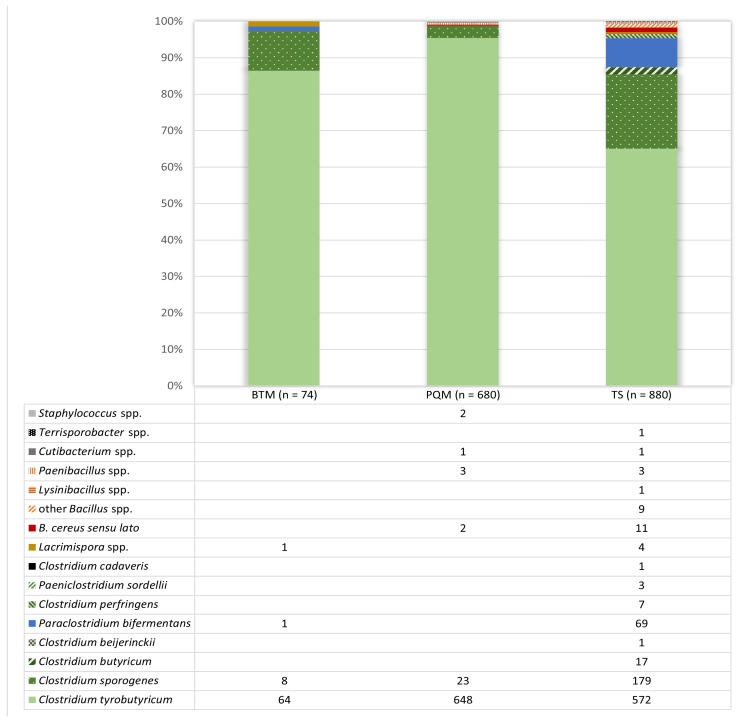
Number of isolates identified per bacterial species by MALDI-TOF MS from positive reactions using the AMP-6000 method used for the analysis of bulk tank milk (BTM), pooled quarter milk (PQM) and teat skin (TS) swab samples.

We were able to obtain 1660 isolates and to identify a total of 1634 isolates from positive reactions in the AMP-6000 method by MALDI-TOF MS. Only isolates identified with a score ≥ 1.7 were included in the results. A fraction of 1.6% of all grown isolates (28 isolates from TS and 8 from PQM samples) remained unidentified, while 84% of the isolates were identified with a score ≥ 2.0 and 93% gave a score ≥ 1.85. Figure 2 provides an overview of the identified isolates per sample type. 

A total of 74 isolates were obtained from bulk tank milk (40 BTM samples), 680 isolates from pooled quarter milk samples (376 PQM samples) and 880 isolates from teat skin swabs (489 TS samples). The majority of isolates from BTM consisted of clostridia (97.2%), with *C. tyrobutyricum* being the predominant species (88.9%) of all BTM isolates, followed by *C. sporogenes* (10.8%). Only two spore-forming genera other than *Clostridium* were identified in BTM: *Lacrimispora* and *Paraclostridium*. However, the detected species have only recently been transferred from the genus *Clostridium* to these new genera [16,17]. Based on presumed genetic similarities and the potential ability to produce gas, we also considered the identified species for further method specificity testing. In PQM, 95.3% of the isolates were identified as *C. tyrobutyricum* and 3.4% as *C. sporogenes*. In PQM, we also sporadically identified the spore-forming genera bacilli and paenibacilli, and a few non-spore-forming cutibacteria and staphylococci. Overall, the results obtained from the analysis of raw milk samples are in good agreement with previous studies that identified 95.7% *Clostridium*, 2.4% other spore-forming genera and 1.9% non-spore-forming species in raw milk [6]. A higher genus and species diversity was observed among isolates from positive reactions when teat skin swabs were analyzed than when milk samples were analyzed. The majority of isolates from teat skin were again assigned to the species considered to be the main causative agent of late blowing in cheese: *C. tyrobutyricum* (65.0%), but also *C. sporogenes* (20.3%), *C. butyricum* (1.9%) and *C. beijerinckii* (0.1%). Interestingly, *Paraclostridium bifermentans* was present in a relatively high abundance (7.8%). This species is considered to be a non-toxigenic commensal that is widely distributed in the farm environment and in the bovine intestine [22]. It has been isolated from cheese and some implications as a potential enhancer of blowing defects in cheese have been proposed [2,23]. Other *Clostridium* species, including the toxigenic *C. perfringens* and *C. cadaveris*, were identified, although only a few isolates. In addition, similar to raw milk, a few former clostridia, now *Lacrimispora* and *Paeniclostridium*, were also identified. Other spore-forming genera were present in small numbers, with the genus *Bacillus* being the most frequently isolated and identified (2.3% of all TS isolates). More than 50% of the isolated bacilli belonged to the *B. cereus* group, emphasizing the high prevalence and relevance of this *Bacillus* group as potential pathogens and/or spoilage agents in milk [24]. However, we refrained from further differentiation of the isolates at the species level due to the limitations of MALDI-TOF MS in distinguishing *B. cereus s.l.* and the limited relevance of detailed discrimination for the purpose of this study [25]. In addition to *B. cereus s.l.*, *B. circulans*, *B. licheniformis*, *B. galactosidilyticus*, *B. pumilus* and *B. oleronius* were identified. Other spore-forming genera included *Paenibacillus*, *Lysinibacillus* and *Terrisporobacter*. *Lysinibacillus* has been isolated from raw milk and milk powder, and some of the isolated strains were able to reduce nitrate and showed toxigenic potential [26,27]. The occurrence of *Paenibacillus* together with clostridia in silage, dairy cow feces and raw milk led Driehuis et al. [28] to conclude that clostridia and paenibacilli share the same contamination sources and the same contamination pathway of milk. This is in good agreement with the results of this study and underlines the need to include paenibacilli as non-target strains in method specificity testing. Most of the aerobic spore-forming species isolated within this study, especially bacilli and paenibacilli, have also been found to be present in raw milk in previous studies [24,29]. However, *Bacillus licheniformis*, although known to be highly prevalent in raw milk, appeared to be inhibited or greatly reduced within the AMPMedia666 used in the present study [24].

The identification of isolates obtained from positive reactions of the AMP-6000 method provided a good overview of the spore-forming and non-spore-forming species that may grow in AMPMedia666 when analyzing samples from the dairy environment. However, it remains unclear whether the identified isolates were the causative agent of the color change in the respective reaction vessel, or whether they may have simply co-occurred with other bacteria that caused the positive reaction in the same vessel. Therefore, based on the species identified from the field samples, a strain library was selected for inclusivity and exclusivity testing with pure bacterial cultures.

### 3.2. Inclusivity of the AMP-6000 Method Based on Pure Culture Testing of C. tyrobutyricum

The results of the inclusivity testing are summarized in Table 1. Out of a total of 45 *C. tyrobutyricum* strains, 40 (88.9%) gave positive results within 48 h using the AMP-6000 method. Of these 40 strains, 34 strains (75.6%) gave quantitative results within the target range of 730–10,000 spores/L milk. Five strains (11.1%) had spore counts > 10,000 spores/L and one strain (2.2%) had a result below the target range with only 223 spores/L (AMP-6000 method). In addition, quantitative results between the alternative (AMP-6000) and the reference method (BB) were in good agreement (statistical details below). Therefore, we confirm the enumeration of these 40 *C. tyrobutyricum* strains using the AMP-6000 method and classify them as inclusive (marked with “I” in Table 1).

**Table 1:** Summary of the results of the inclusivity tests using pure cultures of *Clostridium (C.)*, *Paraclostridium* (P.), *Paeniclostridium (Pa.)* and *Lacrimispora (L.)* in the AMP-6000 method as an alternative method, the reference method using Bryant and Burkey broth (BB) and non-selective plating on RCA. Results are expressed as spores per liter [S/L].

Nr.	Species	Internal/International Strain Number	Source	Alternative MPN: AMP-6000 [S/L]	Reference MPN: BB [S/L]	Spread on RCA [S/L]	Inclusive (I)/Exclusive (E)
1	*C. tyrobutyricum*	Cl 14/DSM 663	Emmental cheese	1550	1400	<1000	I
2	*C. tyrobutyricum*	Cl 82	milk	1460	1700	2000	I
3	*C. tyrobutyricum*	Cl 80	milk	7710	5400	4000	I
4	*C. tyrobutyricum*	Cl 84	milk	2320	9200	3000	I
5	*C. tyrobutyricum*	Cl 188	cheese-sausage mixture	8400	16,000	3500	I
6	*C. tyrobutyricum*	Cl 68	cheddar cheese	7490	>16,000	7500	I
7	*C. tyrobutyricum*	Cl 69	mountain cheese	2020	2400	5500	I
8	*C. tyrobutyricum*	Cl 171	semi-hard cheese	11,368	9200	10,500	I
9	*C. tyrobutyricum*	Cl 83	milk	1827	790	1000	I
10	*C. tyrobutyricum*	Cl 102	milk	10,027	3500	2000	I
11	*C. tyrobutyricum*	Cl 168	semi-hard cheese	16,611	9200	10,000	I
12	*C. tyrobutyricum*	Cl 189	hard cheese	1639	5400	4000	I
13	*C. tyrobutyricum*	Cl 15/DSM 664	raw milk	1827	>16,000	1000	I
14	*C. tyrobutyricum*	Cl 74	milk	3536	1700	4000	I
15	*C. tyrobutyricum*	Cl 86	milk	5012	2800	2500	I
16	*C. tyrobutyricum*	Cl 87	milk	1827	9200	<1000	I
17	*C. tyrobutyricum*	Cl 77	milk	3182	1300	<1000	I
18	*C. tyrobutyricum*	Cl 78	milk	3068	2800	<1000	I
19	*C. tyrobutyricum*	Cl 105	milk	1192	1100	1500	I
20	*C. tyrobutyricum*	Cl 106	milk	2420	1300	1000	I
21	*C. tyrobutyricum*	Cl 107	milk	1021	450	1000	I
22	*C. tyrobutyricum*	Cl 165	mountain cheese	8647	9200	13,000	I
23	*C. tyrobutyricum*	Cl 103	milk	5317	5400	3000	I
24	*C. tyrobutyricum*	Cl 172	Swiss cheese	12,574	9200	5000	I
25	*C. tyrobutyricum*	Cl 174	cheese	3658	3500	1000	I
26	*C. tyrobutyricum*	Cl 175	mountain cheese	223	2400	<1000	I
27	*C. tyrobutyricum*	Cl 182	Gouda cheese	4864	1100	2500	I
28	*C. tyrobutyricum*	Cl 169	mountain cheese	1457	1700	<1000	I
29	*C. tyrobutyricum*	Cl 75	milk	1732	2400	1500	I
30	*C. tyrobutyricum*	Cl 162	milk	1457	1300	2000	I
31	*C. tyrobutyricum*	Cl 167	mountain cheese	8162	3500	4000	I
32	*C. tyrobutyricum*	Cl 16	n.a.	7494	16,000	4000	I
33	*C. tyrobutyricum*	Cl 149	milk	4169	3500	2000	I
34	*C. tyrobutyricum*	Cl 81	milk	4864	9200	1000	I
35	*C. tyrobutyricum*	Cl 88	milk	3068	5400	3000	I
36	*C. tyrobutyricum*	Cl 110	milk	2524	1300	1000	I
37	*C. tyrobutyricum*	Cl 89	milk	2117	2400	7000	I
38	*C. tyrobutyricum*	Cl 90	milk	2317	450	1500	I
39	*C. tyrobutyricum*	Cl 91	milk	3536	1300	1000	I
40	*C. tyrobutyricum*	Cl 178	hard cheese	10,665	2400	14,500	I
41	*C. tyrobutyricum* ^T^	Cl 20/DSM 2637	n.a.	9442	>16,000	11,500	E *
42	*C. tyrobutyricum*	Cl 21/DSM 1460	contaminated culture of *C. kluyveri*	3182	1300	<1000	E *
43	*C. tyrobutyricum*	Cl 33	n.a.	13,511	3500	7500	E *
44	*C. tyrobutyricum*	Cl 180	semi-hard cheese	1457	2400	4500	E *
45	*C. tyrobutyricum*	Cl 85	milk	8162	>16,000	16,000	E *
46	*C. butyricum* ^T^	Cl 19/DSM 10702	pig intestine	531	2400	<1000	I
47	*C. butyricum*	Cl 65	milk	2117	>16,000	1000	I
48	*C. butyricum*	Cl 17/DSM 2477 4P1	cotton tree	2524	>16,000	2000	I
49	*C. butyricum*	Cl 18/DSM 2478 MMP3	lake sediment	9728	>16,000	12,000	I
50	*C. sporogenes*	Cl 2	n.a.	2737	3500	4500	I
51	*C. sporogenes* ^T^	Cl 9/DSM 795	soil	148	690	<1000	I
52	*C. sporogenes*	Cl 60	milk	10,665	>16,000	19,000	I
53	*C. sporogenes*	Cl 12/ATCC 19404	gas gangrene	1732	5400	2500	I
54	*C. sporogenes*	Cl 76	milk	1827	1100	<1000	I
55	*C. sporogenes*	Cl 137	milk	>32,031	>16,000	>900,000	I
56	*C. sporogenes*	Cl 140	milk	>32,031	>16,000	171,000	I
57	*C. beijerinckii*	Cl 49	grass silage	937	9200	16,500	I
58	*C. beijerinckii*	Cl 22	strain R 1357	3182	>16,000	<1000	I
59	*C. beijerinckii*	Cl 24	strain P 1320	73	>16,000	71,500	E
60	*C. beijerinckii*	Cl 46	grass silage	<73	>16,000	11,500	E
61	*C. beijerinckii* ^T^	Cl 36/DSM 791	soil	<73	> 16,000	9500	E
62	*C. beijerinckii*	Cl 48	grass silage	<73	>16,000	32,000	E
63	*C. beijerinckii*	Cl 40/ATCC 6015	n.a.	<73	>16,000	3500	E
64	*C. beijerinckii*	Cl 34/DSM 1820	soil	<73	5400	2000	E
65	*C. beijerinckii*	Cl 44	draff silage	<73	>16,000	2000	E
66	*C. cadaveris*	Cl 67	milk	24,321	1300	15,500	I
67	*C. acetobutylicum* ^T^	Cl 37/DSM 792	cornmeal	2019	>16,000	<1000	I
68	*C. acetobutylicum*	Cl 1/NCDO 1712	n.a.	4864	2400	4500	E*
69	*C. pasteurianum* ^T^	Cl 192/LMG 3285	n.a.	<73	3500	14,000	E
70	*P. bifermentans*	Cl 59	milk	6321	180	12,500	I
71	*P. bifermentans*	Cl 164	milk	5317	>16,000	74,000	I
72	*P. bifermentans*	Cl 133	milk	1732	2400	38,500	I
73	*P. bifermentans*	Cl 126	milk	4169	200	4000	I
74	*P. bifermentans*	Cl 142	milk	12,574	<180	60,000	I
75	*Pa. sordellii* ^T^	Cl 191/LMG 15708	spore appendages	5801	<180	5000	I
76	*L. celerecrescens* ^T^	Cl 194/DSM 5628	culture	<73	200	1500	E

^T^ type strain; n.a. not available, * exclusive within 48 h, inclusive after 72 h incubation.

Strain-dependent differences within the species *C. tyrobutyricum*, which had become apparent in previous phenotypic and genotypic studies, were also observed in the present study. For five *C. tyrobutyricum* strains (Cl 20^T^, Cl21, Cl33, Cl 180 and Cl 85) we observed inhomogeneous color changes from red to yellow after 48 h, which could not be clearly detected by the scanning software and led to inconclusive results. However, after a longer incubation of 72 h instead of 48 h, the color change was also clearly visible and detectable. Nevertheless, since the AMP-6000 readout is made after 48 h, we concluded that these five strains could not be enumerated using the standard AMP-6000 procedure. We defined them to be exclusive with a comment and assigned them an “E *” in Table 1. Interestingly, the type strain Cl20^T^/DSM 2637 was among these exclusive strains. Other studies also reported different phenotypic behaviors between isolates and type strains of *C. tyrobutyricum* [13,30]. For example, the type strain produced more butyric acid in BB medium than strains isolated from Manchego cheese, but less butyric acid in milk than many wild strains [13]. In addition, the type strain produced gas faster than wild strains at 37 °C, while growth at 20 °C was significantly slower than the growth of other strains [30]. Another strain assigned to group E* was strain Cl 21/DSM 1460. Ruusunen et al. also observed that DSM 1460 often behaved differently from strains isolated from cheese or silage in phenotypic studies [31]. The exclusive *C. tyrobutyricum* isolates also include an isolate from semi-hard cheese (Cl 180). Interestingly, this isolate also showed a distinctive genotype (rep-PCR typing) and clustered separately from other *C. tyrobutyricum* strains in a previous study [11]. 

Quantitative data for *C. tyrobutyricum* (*n* = 45) were statistically analyzed. The mean spore counts in log spores/L of raw milk were 3.55 ± 0.38 for the AMP-6000 method, 3.52 ± 0.43 for the BB method and 3.42 ± 0.39 for the RCA method. Previous studies analyzing raw milk samples showed that the BB method yielded results approximately 0.4 log higher than the AMP-6000 method when analyzing the same milk sample [6,10]. However, when pure cultures of *C. tyrobutyricum* were analyzed in the present study, the results of AMP-6000 and BB were not significantly different (*p* = 0.44). When all three methods are compared, RCA results tend to be lower than the AMP-6000 and the BB results, although the Friedman test shows that the observed difference was not significant (*p* = 0.053), indicating good agreement among the three methods. Significant correlations (large effects) were also observed between the results of all tested methods: AMP-6000 and BB (*r_s_* = 0.56, *p* = 0.0002), AMP-6000 and RCA (*r_s_* = 0.60; *p* = 0.0001) and BB and RCA (*r_s_* = 0.52; *p* = 0.0005). These findings support the hypothesis from previous studies that the variation in results when using different methods may be primarily due to the complex microbiota of raw milk samples and the associated selectivity bias [1,5,6].

### 3.3. Inclusivity of the AMP-6000 Method Based on Pure Culture Testing of Clostridium spp.

All *C. butyricum* (*n* = 4) and *C. sporogenes* (*n* = 7) strains tested were quantifiable after 48 h and, therefore, were considered inclusive (I), although we were not always able to stay within the targeted inoculum concentration range, especially due to higher spore levels caused by intense sporulation of *C. sporogenes* isolates. Some *C. butyricum* strains gave high results above the upper detection limit of the BB method, despite lower results in the AMP-6000 and the RCA methods. We hypothesize that a more intense gas production per bacterial cell may be the reason why positive results were seen in the BB method, but growth was reduced in AMPMedia666 and on RCA. *C. butyricum* has been associated with early eye formation in Italian Grana cheese due to its ability to utilize lactose but not lactate. Therefore, it is considered to be less relevant for late blowing defects than *C. tyrobutyricum* [1,32,33,34]. *C. sporogenes* showed intense growth and comparable quantitative results in all media. Therefore, all the tested *C. sporogenes* strains were classified as inclusive. Interestingly, in addition to the color change from red to yellow, the proteolysis of milk proteins by *C. sporogenes* led to clarification of the milk-medium mixture of the AMP-6000 method. This difference may have potential as an additional indicator to differentiate between saccharolytic (e.g., *C. tyrobutyricum*) and proteolytic (e.g., *C. sporogenes*) clostridia. *C. sporogenes* has been isolated from various spoiled cheeses and a differentiation from other *Clostridium* spp. may be interesting as this species is not only known for gas production, but also as a causative agent of spots and blemishes in cheese [13,34,35,36].

Only two out of the 9 *C. beijerinckii* isolates tested (22.2%) gave positive results. The results obtained within this study reveal a weakness of the AMP-6000 procedure for the detection of this *Clostridium* species. This contradicts previous findings where 100% of the *C. beijerinckii* strains (*n* = 13) tested induced the desired color change of the medium, even including Cl 34, Cl 36 and Cl 40, which remained negative within the present study [6]. This can be partly explained by the probably higher inoculum levels and a longer incubation time of 72 h in the previous study compared to 48 h in the present study. Indeed, some endospores germinated when Cl 36 was incubated for 72 h. Nevertheless, for all positive *C. beijerinckii* strains, the spore levels determined by the AMP-6000 method were lower than the spore levels determined by BB and sometimes RCA, again indicating that the species *C. beijerinckii* may be underestimated when using the AMP-6000 method. However, for some isolates (Cl 22, Cl 40 and Cl 44), the results obtained with RCA were also lower than those obtained with the BB method. On the one hand, this may also indicate that detection by gas production in the BB method might overestimate the number of clostridial spores of *C. beijerinckii*. On the other hand, studies have shown that *C. beijerinckii* does not prefer reinforced clostridial medium, which is not only used in the RCA method, but is also used in a modified version in the AMP-6000 method [37]. *C. beijerinckii* has played a minor role in the Austrian dairy industry so far. This species was not, or was very rarely, isolated from Austrian late blown cheeses and Austrian raw milk samples [5,6,38]. However, *C. beijerinckii* is more important in Manchego cheese and it has also been isolated from other cheeses, such as Ossau-Iraty or Beaufort [13,39].

*C. acetobutylicum* DSM 792 was positive in the AMP-6000 method (2019 spores/L) and below the detection limit on RCA (<1000 spores/L), whereas all tubes were positive in the BB method (>16,000 spores/L), indicating a potential overestimation due to the strong hydrogen gas production of this type strain, which is also used for industrial hydrogen production [40]. In contrast, the *C. cadaveris* isolate from milk was positive in all three methods, and high spore counts above 15,000 spores/L were obtained with the AMP-6000 and RCA methods, but gas production in BB appeared to be relatively low compared to cell growth in BB (1300 spores/L). *C. pasteurianum* LMG 3285 was below the lower limit of detection of the AMP-6000 method and was, therefore, considered exclusive (E). This strain also gave lower results in BB (3500 spores/L) than in RCA (14,000 spores/L).

### 3.4. Inclusivity of the AMP-6000 Method Based on Pure Culture Testing of Former Clostridium spp.

Sasi Jyothsna et al. proposed the reclassification of *C. bifermentans* as *Paraclostridium (P.)* within the family Clostridiaceae, and proposed a new genus *Paeniclostridium (Pa.)* to accommodate *C. sordellii* [17]. Since *P. bifermentans* and *Pa. sordellii* have been identified in milk and cheese in previous studies when they were still assigned to the genus *Clostridium* [6,12,15], some strains of these species were also included in the present study. All *P. bifermentans* strains were quantified within a range of 1732 to 12,574 spores/L milk by the AMP-6000 method and were, therefore, considered to be inclusive. However, for the BB and RCA methods, large variations were observed between the test strains. While three strains (Cl 59, Cl 126 and Cl 142) showed results close to the lower limit of detection in the BB method, strong growth up to 60,000 spores/L was observed on RCA, indicating a lack of gas production in the BB medium despite high cell numbers. In contrast, *P. bifermentans* strains Cl 133 and Cl 164 showed moderate and intense gas production in BB and high colony counts on RCA. The role of *P. bifermentans* in relation to late blowing defects is not well understood. Its repeated isolation from the dairy environment and potential gas production suggests that this species merits further investigation in relation to late-blown cheese. Even fewer data are available on the species *Pa. sordellii* in the context of cheese spoilage. The type strain tested in this study induced a color change in the AMP-6000 method and grew on RCA with comparable results between AMP-6000 and RCA of approximately 5000 spores/L milk. Therefore, this strain was considered to be inclusive. *Pa. sordellii* strain Cl 191 could not be quantified using the BB method because no gas production was observed.

As *Clostridium celerecrescens*/*Desulfotomaculum guttoideum* was also isolated from spoiled cheese in a previous study [38], we also included the type strain of this species in our test strain set, but now under the name *Lacrimispora celerecrescens* due to the species reclassification in 2020 [16]. The type strain of this species did not induce color change in the AMP-6000 method and is, therefore, considered to be exclusive. DSM 5628 also showed only weak gas production in the reference method, while sufficient growth was confirmed on RCA.

In summary, out of 76 strains tested, 61 were inclusive (I), 6 were inclusive after prolonged incubation (E *) and 9 strains were exclusive (E).

### 3.5. Exclusivity of the AMP-6000 Method Based on Pure Culture Testing

The results of the exclusivity testing of spore-forming bacteria are summarized in Table 2. A strain was classified as “exclusive” if the results were below the detection limit in the AMP-6000 method, but at the same time sufficient inoculum and growth were confirmed in the non-selective RCA method. One of the most important spore-forming genera in raw milk is *Bacillus*, and among the bacilli, members of the *Bacillus cereus* group or *B. cereus s.l.* are particularly relevant in dairy foods [24].

**Table 2:** Summary of the results of the exclusivity testing using pure cultures of spore-forming bacteria in the AMP-6000 method as an alternative method, the reference method using Bryant and Burkey broth (BB) and non-selective plating on RCA under anaerobic and aerobic conditions. Results are expressed as spores per liter [S/L].

Nr.	Species	Internal/ International Strain Number	Alternative MPN: AMP-6000 [S/L]	Reference MPN: BB [S/L]	Spread on RCA (37 °C, Anaerobic) [S/L]	Spread on RCA (30 °C, Aerobic) [S/L]	Inclusive (I)/Exclusive (E)
1	*Bacillus cereus* ^T^	DSM 31	<73	<4100 ^3^	3.0 × 10^6^	2.0 × 10^6^	E
2	*Bacillus cereus*	LMG 8221	<73	<4100 ^3^	1.4 × 10^6^	1.6 × 10^5^	E
3	*Bacillus cereus*	LMG 12,334	<73	<4100 ^3^	5.0 × 10^5^	7.3 × 10^5^	E
4	*Bacillus cereus*	ATCC 7004	<73	<4100	1.9 × 10^9^	1.4 × 10^9^	E
5	*Bacillus cereus s.l.*	I-Bc 1 (milk)	<73	<4100	1.4 × 10^9^	1.9 × 10^9^	E
6	*Bacillus cytotoxicus* ^T^	LMG 26,718	<73	<4100 ^3^	3.6 × 10^5^	7.6 × 10^5^	E
7	*Bacillus thuringiensis* ^T^	LMG 7138	<73	<4100	6.0 × 10^5^	6.3 × 10^5^	E
8	*Bacillus mycoides* ^T^	LMG 7128	<73	<4100	<1.0 × 10^5^	>3.0 × 10^6^	E
9	*Bacillus pseudomycoides*	LMG 18,993	<73	<4100 ^3^	1.2 × 10^5^	1.4 × 10^4^	E
10	*Bacillus wiedmannii* ^T^	LMG 29,269	<73	<4100 ^3^	8.7 × 10^5^	9.7 × 10^5^	E
11	*Bacillus mycoides* ^T,2^	DSM 11,821	<73	<4100 ^3^	<1.0 × 10^4^	1.4 × 10^6^	E
12	*Bacillus licheniformis* ^T^	DSM 13	<73	<4100 ^3^	2.4 × 10^5^	3.3 × 10^5^	E
13	*Bacillus sonorensis* ^T^	LMG 21,636	<73	<4100 ^3^	1.2 × 10^5^	9.4 × 10^5^	E
14	*Bacillus sonorensis*	I-Bc 3 (milk)	<73	<4100	>3.0 × 10^1^	>3.0 × 10^10^	E
15	*Bacillus oleronius* ^T^	LMG 17,952	<73	<4100 ^3^	<1.0 × 10^4^	3.5 × 10^4^	E
16	*Bacillus oleronius*	I-Bc 2 (teat skin)	<73	<4100	<1.0 × 10^5^	1.1 × 10^10^	E
17	*Bacillus galactosidilyticus* ^T^	LMG 17,892	<73	<4100	<1.0 × 10^5^	2.8 × 10^7^	E
18	*Bacillus galactosidilyticus*	I-Bc 4 (milk)	<73	<4100	<1.0 × 10^5^	5.6 × 10^8^	E
19	*Bacillus pumilus* ^T^	LMG 18,928	<73	<4100	<1.0 × 10^5^	1.1 × 10^7^	E
20	*Bacillus pumilus*	I-Bc 5 (milk)	<73	<4100 ^3^	<1.0 × 10^4^	4.3 × 10^5^	E
21	*Bacillus subtilis* ssp. *subtilis* ^T^	LMG 7135	<73	<4100	>3.0 × 10^1^	>3.0 × 10^10^	E
22	*Bacillus subtilis* ssp. *spizizenii* ^T^	DSM 15,029	<73	<4100 ^3^	<1.0 × 10^4^	5.7 × 10^5^	E
23	*Lysinibacillus sphaericus* ^T^	LMG 7134	<73	<4100	<1.0 × 10^5^	>3.0 × 10^10^	E
24	*Lysinibacillus sphaericus*	I-LBc 1 (teat skin)	<73	<4100	<1.0 × 10^5^	>3.0 × 10^10^	E
25	*Paenibacillus lactis* ^T^	LMG 21,910	<73	<4100 ^3^	<1.0 × 10^4^	7.7 × 10^4^	E
26	*Paenibacillus lactis*	I-Pa 1 (milk)	<73	<4100	8.0 × 10^9^	1.3 × 10^10^	E
27	*Paenibacillus macerans* ^T^	LMG 6324	<73	>11,000	2.4 × 10^6^	1.7 × 10^6^	E
28	*Paenibacillus macerans*	DSM 24,746	<73	>11,000	8.8 × 10^4^	3.0 × 10^5^	E
29	*Paenibacillus odorifer* ^T^	LMG 19,079	<73	<4100	9.9 × 10^9^	1.4 × 10^10^	E
30	*Paenibacillus peoriae* ^T^	LMG 14,832	<73	<180 ^3^	4.2 × 10^5^	<1.0 × 10^5^	E
31	*Paenibacillus barengoltzii* ^T^	DSM 22,255	<73	<180 ^3^	<1.0 × 10^5^	7.7 × 10^6^	E
32	*Paenibacillus pabuli* ^T^	LMG 15,970	<73	<4100	<1.0 × 10^5^	8.5 × 10^7^	E
33	*Paenibacillus polymyxa* ^T^	LMG 13,294	<73	1300	1.9 × 10^3^	5.2 × 10^3^	E
34	*Paenibacillus glucanolyticus* ^T^	LMG 12,239	<73	<4100 ^3^	5.0 × 10^4^	6.4 × 10^4^	E
35	*Paenibacillus amylolyticus* ^T^	LMG 14,012	<73	<4100	1.6 × 10^8^	2.2 × 10^9^	E
36	*Paenibacillus lautus* ^T^	LMG 11,157	>32,000	<4100	8 × 10^9^	9.7 × 10^8^	I
37	*Paenibacillus lautus*	LMG 14,669	>1732	<4100	6.2 × 10^5^	7.3 × 10^5^	I

^T^ type strain; ^2^ originally deposited as type strain of *B. weihenstephanensis*; ^3^ gas bubbles observed under paraffin plug.

### 3.6. Exclusivity of the AMP-6000 Method Based on Pure Culture Testing of Bacillus spp.

None of the 11 *B. cereus sensu lato* strains tested in this study induced a color change in the AMP-6000 method, and results were below the detection limit (<73 spores/L) despite high inoculum concentrations of >10^5^ spores/L milk. Furthermore, no *B. cereus s.l.* strain produced gas in the BB medium of the reference method, indicating that this non-target group was also excluded in the reference method.

*B. licheniformis* (*n* = 1) and *B. sonorensis* (*n* = 2) strains were also excluded in the AMP-6000 method and the BB method as no color change or gas production was observed. *B. oleronius* (*n* =1), *B. galactosidilyticus* (*n* = 2) and *B. pumilus* (*n* = 2) also gave results below the detection limits of the AMP-6000 and the BB method despite high inoculum levels. Interestingly, the lack of growth of these strains on RCA under standard incubation conditions for clostridia (37 °C, anaerobic) and good growth on RCA under aerobic conditions at 30 °C indicate that these strains are successfully excluded from the AMP-6000 and the BB analysis by the chosen anaerobic incubation conditions. Both *B. subtilis* strains were also confirmed as non-target strains of the AMP-6000 and the BB method, as the results were below the detection limits of these methods. In summary, 100% of the 22 bacilli tested were negative and, therefore, excluded by both the AMP-6000 method and the reference (BB) method.

### 3.7. Exclusivity of the AMP-6000 Method Based on Pure Culture Testing of Lysinibacillus and Paenibacillus

The type strain of *Lysinibacillus sphaericus* and a milk isolate were also negative in both the AMP-6000 and the BB method. Heterogeneities were observed among the test strains of the genus *Paenibacillus*. Of the 13 paenibacilli, 11 gave results below the detection limit of the AMP-6000 method and were, therefore, confirmed as exclusive. Interestingly, among these eleven strains, two *P. macerans* strains showed strong gas production in the BB method, indicating that this species may bias clostridial quantification when the BB method is used, but not when the AMP-6000 method is applied. This is in agreement with findings from Driehuis et. al., who observed gas production by *P. macerans* in Van Beynum and Pette medium [28]. In contrast, *P. lautus* (*n* = 2) was not detected by the BB method, but gave positive results with the AMP-6000 method, indicating a weakness of the AMP-6000 method to exclude *P. lautus*.

For some bacilli and paenibacilli, weak gas production in the form of bubbles under the paraffin plug was also observed in one or more tubes of the BB method (strains are indicated in Table 2), but the amount of gas was not sufficient to lift the plug completely from the culture medium. Therefore, these results were considered negative. However, when pea-sized bubbles are interpreted as positive results in some laboratories, the interference of gas-producing bacilli and paenibacilli and potential false positive results should be considered.

### 3.8. Exclusivity of the AMP-6000 Method Based on Pure Culture Testing of Lactic Acid Bacteria

As the AMP-6000 method has also been used to enumerate clostridial spores in cheese or in cheese milk containing starter cultures [41], we also investigated whether lactic acid bacteria in high numbers would survive pasteurization and potentially induce a color change in AMPMedia666. For this purpose, in addition to the 37 spore-forming strains, we tested the type strains of 14 lactic acid bacteria, including typical species commonly involved in dairy fermentation. Detailed species and strain information and a summary of the results are shown in Table 3.

**Table 3:** Summary of the results of the exclusivity testing using pure cultures of lactic acid bacteria in the AMP-6000 method as an alternative method, the reference method using Bryant and Burkey broth (BB) and non-selective plating on RCA under anaerobic and aerobic conditions. Results are expressed as spores per liter [S/L] or colony forming units per liter [CFU/L].

Nr.	Species	Internal/ International Strain Number	Alternative MPN: AMP-6000 [S/L]	Reference MPN: BB [S/L]	Spread on RCA (37 °C) [CFU/L] (Pasteurized)	Spread on MRS [CFU/L] (Optimum Growth Conditions; Unpasteurized)	Inclusive (I)/Exclusive (E)
1	*Lactobacillus amylovorus* ^T^	DSM 20531	<73	<3700	<1.0 × 10^4^	4.2 × 10^10^	E
2	*Lacticaseibacillus rhamnosus* ^T^	DSM 20021	<73	<3700	<1.0 × 10^4^	1.1 × 10^10^	E
3	*Lactobacillus delbrueckii* ssp. *bulgaricus* ^T^	DSM 20081	<73	<3700	<1.0 × 10^4^	8.1 × 10^9^	E
4	*Lentilactobacillus buchneri* ^T^	DSM 20057	<73	<3700	<1.0 × 10^4^	1.3 × 10^10^	E
5	*Limosilactobacillus fermentum* ^T^	DSM 20052	<73	<3700	<1.0 × 10^4^	4.0 × 10^10^	E
6	*Lactiplantibacillus plantarum* ^T^	DSM 20174	<73	<3700	<1.0 × 10^4^	7.8 × 10^10^	E
7	*Lactobacillus acidophilus* ^T^	DSM 20079	<73	<3700	<1.0 × 10^4^	5.7 × 10^9^	E
8	*Lentilactobacillus kefiri* ^T^	DSM 20587	<73	<3700	<1.0 × 10^4^	1.3 × 10^10^	E
9	*Enterococcus faecalis* ^T^	DSM 20478	<73	<3700	<1.0 × 10^4^	6.1 × 10^9^	E
10	*Enterococcus durans* ^T^	DSM 20633	<73	<3700	<1.0 × 10^4^	1.9 × 10^9^	E
11	*Lactococcus lactis* ^T^	DSM 20481	<73	<3700	1.1 × 10^4^	6.8 × 10^10^	
12	*Leuconostoc lactis* ^T^	DSM 20202	<73	<3700	<1.0 × 10^4^	1.5 × 10^10^	E
13	*Leuconostoc mesenteroides* ssp. *mesenteroides* ^T^	LMG 6893	<73	<3700	<1.0 × 10^4^	7.5 × 10^10^	E
14	*Streptococcus salivarius* ssp. *thermophilus* ^T^	LMG 6896	<73	<3700	7.9 × 10^5^	3.5 × 10^9^	E

^T^ type strain.

On non-selective RCA, *Streptococcus salivarius* ssp. *thermophilus* LMG 6896 showed growth even after pasteurization, and a few colonies of *Lactococcus lactis* DSM 20481 were also counted on RCA. However, these strains remained negative in both the AMP-6000 method and the BB methods. In fact, despite high inoculum levels of 10^9^ to 10^10^ prior to pasteurization, all 14 LAB strains tested gave results <73 spores/L in the AMP-6000 method when the sample had been pasteurized. None of the strains tested produced gas in the BB method either, confirming successful pasteurization and no interference with clostridial enumeration also in the BB method.

### 3.9. Discrepancies between Quantitative Results of the Three Test Methods—General Considerations

The quantitative results of the three test methods were sometimes contradictory, even when the same pure bacterial culture was analyzed. Differences have been observed previously when different methods were used for raw milk analysis and can be partly explained by differences in media composition, methodological procedures, incubation and detection principles [3,5,6,10,34]. In the present study, we were able to show that, for the majority of pure cultures of *C. tyrobutyricum* tested, no significant difference was observed between quantitative results of the AMP-6000 and the BB methods. However, we also discovered a diversity in growth behavior, color change and gas production, especially among strains other than *C. tyrobutyricum*, thereby shedding more light on strain- and species-dependent biases that may be introduced by the different members of the raw milk microbiota. Strain- and species-dependent variations, and consequent inconsistencies between different enumeration methods, might be particularly relevant when very low spore counts (e.g., <2 log spores/L of cheese milk) can cause severe blowing defects [38,42]. This is often the case for traditional Austrian, Swiss or Italian hard cheeses [34,38,43]. To prevent late blowing in these cheeses, barn and feed hygiene, as well as technological measures during cheese production, are effective strategies to keep clostridial spore levels in raw milk as low as possible [1,4,8,44]. Finally, monitoring clostridial spore levels and understanding the advantages and limitations of the analytical method used to monitor spore levels when interpreting the results may be essential for optimizing mitigation strategies and ensuring cheese milk quality.

## 4. Conclusions

The AMP-6000 method was found to be suitable for the quantification of the majority of *C. tyrobutyricum*, and all *C. butyricum* and *C. sporogenes* strains tested. *C. beijerinckii*, which is also associated with late blowing and has been isolated from spoiled Manchego cheese, may be underestimated by the AMP-6000 method. However, former *Clostridium* species that have been reclassified as *Paraclostridium* and *Paeniclostridium* were detected and their role in cheese spoilage requires further investigation. All non-target strains of the tested genera *Bacillus*, *Lysinibacillus* and *Paenibacillus* were confirmed negative, with the exception of *Paenibacillus lautus*. Furthermore, we have shown that lactic acid bacteria do not interfere with the detection of clostridial spores in raw milk, even when present in high numbers in milk prior to pasteurization. Overall, the quantitative results of the three test methods were sometimes contradictory when analyzing the same pure culture. This underlines the importance of taking into account the different methods and detection principles when interpreting the results, which is particularly relevant when clostridial spore counts in milk are considered for quality payments.

## Data Availability

The original contributions presented in the study are included in the article, further inquiries can be directed to the corresponding author.
